# Encapsulation of Green Tea Extract (GTE) in Nanoliposome and Assessment of Its Characterization and In Vitro Release Study of GTE

**DOI:** 10.1002/fsn3.70781

**Published:** 2025-08-16

**Authors:** Nadia Ahmadi, Parham Joolaei Ahranjani, Ladan Rashidi

**Affiliations:** ^1^ Food Technology and Agriculture Products Research Center Standard Research Institute (SRI) Karaj Iran; ^2^ Faculty of Agricultural, Environmental and Food Sciences Free University of Bolzano‐Bozen Bolzano Italy

**Keywords:** antioxidant efficacy, bioavailability, encapsulation, green tea extract (GTE), nanoliposome

## Abstract

This study aimed to encapsulate GTE in liposomes using a thin‐film ultrasonic dispersion method to enhance its stability and bioavailability. The optimal formulation used a tea polyphenol‐to‐lecithin ratio of 0.125:1, lecithin‐to‐cholesterol ratio of 4:1, and PBS at pH 6.62, yielding liposomes with an encapsulation efficiency (EE) of 60.09%, corresponding to the optimized formulation (Sample T25: phosphatidylcholine:cholesterol ratio of 2:1, 0.6% Tween 80, and 1000 ppm GTE), selected for its balance of size, stability, and performance, a particle size of 99.2 ± 0.34 nm, and a zeta potential of approximately −30 mV. Antioxidant activity, measured by the DPPH assay, showed that encapsulation significantly improved the free radical scavenging ability of green tea polyphenols. Additionally, release kinetics in simulated gastric (SGF) and intestinal fluids (SIF) followed a biphasic profile, with an initial burst release followed by sustained release, fitting best with the Korsmeyer–Peppas model. Stability studies demonstrated that the liposomal formulation maintained consistent particle size and EE over 90 days, confirming its ability to preserve GTE's bioactivity under simulated physiological conditions. These findings emphasize the potential of liposome‐encapsulated green tea polyphenols as an effective delivery system, offering enhanced stability and bioavailability for food and therapeutic applications. The study provides insights into optimizing liposomal formulations for the delivery of bioactive compounds, supporting their use in functional foods and nutraceuticals.

## Graphical abstract

This study successfully encapsulated green tea extract in liposomes through a thin film ultrasonic dispersion method, achieving optimal conditions that resulted in high encapsulation efficiency and stability, while enhancing bioavailability and protecting the potent health benefits of polyphenols like EGCG for various applications.

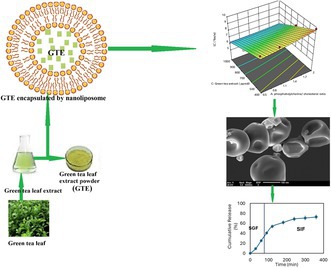

## 1 Introduction

Green tea has received considerable attention due to its wide array of health benefits, which encompass the reduction of cholesterol levels, antibacterial and antiviral properties, as well as the prevention of cancer and cardiovascular diseases. Additionally, it exhibits anti‐inflammatory and antiarthritic effects (Ahmadi et al. 2024; Jabeen et al. 2022; Sunna et al. 2019). Recent studies have further emphasized its efficacy against various microorganisms, including fungi and bacteria, thereby indicating its potential role in promoting oral health (Potocka et al. 2023). The health‐enhancing properties of green tea are primarily attributed to the combined effects of its numerous constituents, especially epigallocatechin gallate (EGCG), rather than the isolated effects of individual components (Ahmadi et al. 2024; Sahadevan et al. 2023).

The significant antioxidant properties of green tea can be attributed to its abundant polyphenolic compounds, which encompass flavonols and catechins. These polyphenols act as scavengers of free radicals, thereby playing an essential role in the prevention of lipid oxidation and potentially improving the shelf life of food items (Gutiérrez‐Del‐Río et al. 2021; Ma et al. 2024; Zhang et al. 2021). The variety of bioactive compounds found in green tea, including flavanols, flavandiols, and flavonoids, is crucial not only for their antioxidant capabilities but also for their roles in cancer prevention and anti‐inflammatory responses (Bhuvaneswari et al. 2023; Jha and Pathak 2023; Jara‐Quijada et al. 2024). To enhance the stability and bioavailability of these antioxidant substances, the method of encapsulation within liposomes has been suggested. This strategy aims to shield the bioactive components from environmental factors, facilitating their controlled release and preserving their effectiveness (Maqsoudlou et al. 2020; Sabaghi et al. 2021; Jara‐Quijada et al. 2024). However, green tea catechins, particularly EGCG, are known to be unstable in neutral and alkaline pH conditions, susceptible to oxidative degradation, and exhibit low systemic bioavailability due to short biological half‐life and poor membrane permeability (Radeva‐Ilieva et al. 2025). The encapsulation technique is gaining recognition in the food sector for its potential to improve the functional and nutritional attributes of products by protecting natural antioxidants (Sabaghi et al. 2021).

The rapid degradation and poor intestinal absorption of tea polyphenols present significant challenges; however, encapsulation technology provides a viable solution to enhance their stability and bioavailability (Rashidinejad et al. 2021; Grgić et al. 2020). By encapsulating active compounds within a protective matrix, this technology acts as a barrier that helps maintain the bioactivity of sensitive substances (Sheybani et al. 2023a; Ekrami et al. 2022; Pateiro et al. 2021). A variety of encapsulation techniques, including coacervation, electrospinning, emulsification, and anti‐solvent precipitation, have been investigated to improve the effectiveness and applicability of bioactive compounds in the food and agricultural sectors (Faraji Sarabmirza et al. 2023; Gopi et al. 2022; Fawzy et al. 2024). The application of liposomes for the delivery of phenolic compounds represents an innovative strategy for stabilizing oil formulations and integrating dietary docosahexaenoic acid (DHA), which may play a role in disease prevention (Wang et al. 2021).

This research focused on the formulation of liposome‐encapsulated GTE and assessed the efficacy of GTE encapsulated in liposomes as a natural antioxidant, comparing its effectiveness to that of conventional antioxidants while also examining the opportunities and challenges posed by the complex phenolic composition of green tea in the development of derivative products. Ultimately, the study evaluated the limitations concerning the stability and bioavailability of green tea polyphenols achieved through encapsulation. This study uniquely applies multivariate optimization using Box–Behnken design and assesses long‐term encapsulation efficiency and particle size stability over 90 days. Moreover, FTIR was used to confirm storage‐induced structural preservation, and biphasic release kinetics were characterized using the Korsmeyer–Peppas model, all of which collectively distinguish this study from prior literature.

The investigation into liposome‐encapsulated GTE provides significant understanding regarding the formulation and efficacy of natural antioxidants relative to traditional alternatives.

## 2 Materials and Methods

### 2.1 Material

The GTE used in this study was obtained from *Camellia sinensis* (*Theaceae* family) and sourced from Komin, Ireland. Soy lecithin (Lipoid S75 with 80% phosphatidylcholine) was supplied by Lipoid GmbH, Ludwigshafen, Germany. Cholesterol (1000 IU/g), a dialysis bag with a molecular weight cutoff of 12,000 Da, and the Folin–Ciocalteu reagent, which is essential for the analysis of phenolic content, were procured from Sigma‐Aldrich, Germany. Ethanol used in the preparation processes was obtained from Carlo Erba, France. All other reagents and chemicals utilized in this study were of analytical grade, purchased from Merck, Germany, to ensure the highest quality and reliability of the experimental outcomes.

### 2.2 Liposome Preparation

Liposomes containing GTE were synthesized through the ethanol injection technique. The process commenced with the preparation of a lipid solution, wherein soy phosphatidylcholine (SPC) was dissolved at a concentration of 15 μmol lipid/mL, and cholesterol was incorporated at a ratio of 4:1 relative to SPC, along with GTE in 5 mL of ethanol (Carlo Erba, France). This ethanol solution was then gradually added to 70 mL of distilled water while being vigorously stirred using a homogenizer (Heidolph, Germany) set at 20,000 rpm, facilitating the effective dispersion and integration of the liposomal constituents (Shah et al. 2020). Although the initial steps involve ethanol dissolution, the subsequent formation of a dry lipid film via methanol/dichloromethane evaporation and rehydration followed by probe sonication corresponds to the thin‐film ultrasonic dispersion technique. This hybridized approach was selected to enhance bilayer uniformity, optimize GTE entrapment, and reduce vesicle size. To create nanoliposomes with distinct properties, various lecithin‐to‐cholesterol ratios (0–60, 30–30, 40–20, and 20–40 mg) were investigated. A thin lipid film was produced by dissolving GTE at different concentrations in a 1:1 mixture of methanol and dichloromethane, followed by evaporation at 30°C using a rotary evaporator under vacuum conditions. Following this, 20 mL of deionized water containing the optimal methanol extract was introduced to the lipid residue. The resulting mixture underwent rotary evaporation under vacuum at 30°C for 30 min, enabling the lipid phase to dissolve into the aqueous phase and yielding multilayered, micrometric liposomes (De Leo et al. 2022). To transform these liposomes into a single‐layer nanoscale structure, a sonication process was utilized. The samples were sonicated with a probe sonicator, employing cycles of 1 min of sonication followed by a 1‐min pause, repeated as necessary to ensure the uniformity and stability of the nanoliposomes (Khuntia et al. 2022).

### 2.3 Encapsulation Efficiency (EE)

The encapsulation efficiency (EE) of phenolic compounds within liposomes was calculated as a percentage using the following equation (Equation 1):

(1) $$\mathrm{EE}\left(\%\right)=\left(\frac{C_{\text{total}}-C_{\text{free}}}{C_{\text{total}}}\right)\times 100$$

where *C*_total_ represents the total concentration of phenolic compounds released after liposome disruption with methanol, and *C*_free_ denotes the concentration of phenolic compounds that were not encapsulated, as determined from the filtrate collected using a Millipore filtration system or from the supernatant (Sabaghi et al. 2021). The quantification of phenolic compounds in liposomes involved the preparation of a gallic acid calibration curve, followed by the separation of free and encapsulated phenolics. The aqueous phase containing free phenolics was mixed with sodium carbonate and Folin–Ciocalteu reagent, centrifuged, and the absorbance of the supernatant was measured at 750 nm (Yousefi et al. 2023).

### 2.4 Mean Particle Size Measurement

The mean particle size and polydispersity index (PDI) of the nanoliposome encapsulating green tea extract (GTE) were assessed through dynamic laser light scattering (DLS) utilizing a particle size analyzer (Mastersizer 3000, Malvern Panalytical Ltd., UK). This evaluation aimed to identify the optimal characteristics of the liposomes, particularly those exhibiting enhanced encapsulation efficiency (EE), both prior to and following the encapsulation of GTE (Jara‐Quijada et al. 2024).

### 2.5 Zeta Potential (ZP) Measurement

The zeta potential (ZP) of nanoliposomes containing GTE was measured using the laser Doppler electrophoretic (LDE) technique with a Zetasizer (Mastersizer 3000, Malvern Panalytical Ltd., UK), equipped with a helium‐neon laser (*λ* = 630 nm) at 25°C (Rashidi et al. 2017).

### 2.6 Fourier Transform Infrared Spectroscopy (FT‐IR) Analysis

Fourier Transform Infrared Spectroscopy (FT‐IR) was employed using a Perkin Elmer spectrometer to analyze the molecular interactions between water‐soluble compounds in GTE and the lipid membranes of nanoliposomes. Post‐lyophilization samples were analyzed in transmittance mode using potassium bromide (KBr) disks over a wavenumber range of 400–4000 cm^−1^. The sample thickness was maintained between 0.01 and 0.1 mm to ensure precise spectral data acquisition (Rashidi et al. 2017).

### 2.7 Morphological Characterization

The morphology of the GTE nanoliposomes was examined using Scanning Electron Microscopy (SEM) (FEI Inspect S50 CAE, USA), which provided detailed insights into the particle structure and encapsulation integrity. For SEM analysis, samples were mounted on steel supports using carbon tape, followed by sputter‐coating with a thin gold layer to enhance conductivity. The prepared samples were then exposed to an accelerated electron beam at an excitation voltage of 10 kV, enabling high‐resolution imaging of both external and internal characteristics of the nanoliposomes (Shariare et al. 2020).

### 2.8 Evaluation of Liposomal Permeability

The permeability of GTE‐encapsulating liposomes was quantitatively assessed by calculating the EE% at different storage durations (1, 15, 30, 60, and 90 days). The permeability percentage (*P*%) was determined using the equation (Equation 2):

(2) $$P\left(\%\right)=\left(\frac{{\mathrm{EE}}_{a}-{\mathrm{EE}}_{b}}{{\mathrm{EE}}_{a}}\right)\times 100$$

where EE_*a*_ is the encapsulation efficiency immediately after liposome preparation (day 1), and EE_*b*_ represents the encapsulation efficiencies observed after storage, respectively. This method allows for the evaluation of the liposomal system's ability to retain GTE over time, indicating the stability and permeability properties of the formulation (Jahanfar et al. 2021).

### 2.9 In Vitro Release Kinetic Study

The evaluation of phenolic compound release from GTE‐encapsulated liposomes was conducted using in vitro simulated gastric fluid (SGF) at a pH of 2.3 and a temperature of 37°C for a duration of 2 h, followed by exposure to simulated intestinal fluid (SIF) at a pH of 7.4 and the same temperature for an additional 4 h (Iraji et al. 2018). A total of 10 mg of GTE‐encapsulated liposomes was placed within a dialysis bag, which was initially submerged in 50 mL of SGF at pH 2.3 for 2 h. Subsequently, the bag was transferred to 60 mL of SIF at pH 7.4 for 4 h, maintaining a constant temperature of 37°C and stirring at 100 rpm. Samples from the surrounding medium were collected at specified time intervals (0, 30, 60, 90, 120, and 180 min for SGF; 240, 300, and 360 min for SIF), with immediate replenishment of the medium volume. The concentration of released GTE was quantified using spectrophotometry at a wavelength of 297 nm. To investigate the release kinetics, four mathematical models were employed: zero‐order (Equation 3), first‐order (Equation 4), Higuchi (Equation 5), and Korsmeyer–Peppas (Equation 6). These models facilitated the identification of the most appropriate release mechanism for GTE, based on the correlation coefficient (*R* ^2^) between the observed and predicted values.

(3) $$\frac{M_{t}}{M_{\infty }}=\mathrm{Kt}$$

(4) $$\ln M_{t}=\ln M_{\infty }-\mathrm{Kt}$$

(5) $$\frac{M_{t}}{M_{\infty }}=K\sqrt{t}$$

(6) $$\frac{M_{t}}{M_{\infty }}=\mathrm{Ktn}$$

where *k* is the release rate constant, *t* is time, *n* is the release exponent, and *M*_*t*_ and *M*_*∞*_ represent the amounts of GTE released at time *t* and at infinite time, respectively. The release exponent “*n*” helps categorize the release mechanism as Fickian diffusion (*n* ≤ 0.45), non‐Fickian transport (0.45 < *n* < 0.89), or erosion‐controlled release (*n* ≥ 0.89) (Iraji et al. 2018). This comprehensive analysis provides a detailed understanding of the release dynamics of GTE from nanoliposomes under simulated physiological conditions.

### 2.10 Assessment of Physical Stability in Nanoliposomes Encapsulating GTE

The physical stability of nanoliposomes containing GTE was evaluated by storing the formulations under refrigerated conditions and systematically assessing critical stability metrics. These metrics included particle size, surface morphology analyzed through scanning electron microscopy (SEM) and encapsulation efficiency (EE%). Monitoring of these parameters occurred over a 90‐day duration to determine the physical integrity and stability of the formulations throughout extended storage. This evaluation offered valuable insights into the impact of prolonged storage on the structural and functional characteristics of the nanoliposomes, thus aiding in the estimation of their shelf life and efficacy (Monasterio and Osorio 2024).

### 2.11 Statistical Analysis

All experiments were conducted in triplicate, and the results are presented as the mean ± standard deviation. Statistical analysis was performed using Analysis of Variance (ANOVA), with significant differences among means evaluated using Duncan's multiple range test at a 95% confidence level (*p* < 0.05).

## 3 Results and Discussion

### 3.1 Optimization Model

EE serves as a vital parameter in the formulation of liposomes, especially in the context of enhancing the design of GTE‐loaded liposomes. The optimization of these formulations was conducted utilizing a statistical model based on Response Surface Methodology (RSM). The empirical relationship among the variables, specifically the ratio of phosphatidylcholine‐to‐cholesterol (A), the concentration of Tween 80 (B), and the concentration of GTE (C), in relation to the response variable EE% is articulated in Equation (7). This equation integrates both linear and quadratic effects to improve the model's efficacy (*p* < 0.05) in achieving optimal EE%. The results of the ANOVA analysis for this optimization model are presented in Table 1a.

(7) $$\begin{array}{cc} \mathrm{EE}= & 79.73288+6.68544x_{1}+47.44951x_{2}-0.035774x_{3}+\\ & 5.13951x_{1}x_{2}-0.011924x_{1}x_{3}+0.003101x_{2}x_{3}+\\ & 1.66244{x_{1}}^{2}-69.89456{x_{2}}^{2}+4.89756E-06{x_{3}}^{2} \end{array}$$

**TABLE 1a:** Analysis of variance (ANOVA) and regression coefficients for the quadratic model of EE%.

Sources of variance	Mean squares	Degrees of freedom	Sum of squares	*F*‐value	*p*
Model	3411.41	9	379.05	35.97****	> 0.0001
A: phosphatidylcholine: cholesterol ratio	176.27	1	176.27	16.73***	0.0008
B: Tween 80	55.06	1	55.06	5.23*	0.0354
C: GTE	2861.28	1	2861.28	271.55****	> 0.0001
AB	9.91	1	9.91	0.9404^ns^	0.3458
AC	87.37	1	87.37	8.29*	0.0104
BC	0.6847	1	0.6847	0.0650^ns^	0.8018
A^2^	3.93	1	3.93	0.3726^ns^	0.5497
B^2^	100.65	1	100.65	9.55**	0.0066
C^2^	1.02	1	1.02	0.0965^ns^	0.7598
Residual	179.13	17	10.54		
Lack of fit	166.30	14	11.88	2.78^ns^	0.2171
Pure error	12.83	3	4.28		
Cor total	3590.53	26			
	*R* ^2^	0.96			
	Predicted *R* ^2^	0.88			

*Note:* ns = not significant; *p* < 0.05 (*), *p* < 0.01 (**), *p* < 0.001 (***), *p* < 0.0001 (****).

RSM was utilized to investigate the impact of various factors, specifically the concentrations of Tween 80 and the ratio of phosphatidylcholine to cholesterol, on the encapsulation efficiency (EE%). To optimize EE% while enhancing the response, a Box–Behnken Design (BBD) was employed. A total of 27 batches of nanoliposomes were produced, varying three independent variables: A (the ratio of phosphatidylcholine to cholesterol), B (the concentration of Tween 80), and C (the concentration of GTE). As detailed in Table 1a, the statistical significance of the model developed through RSM was assessed using the *F*‐value and *p*‐value, with a *p*‐value of less than 0.05 indicating that the model terms were statistically significant. The quadratic model for EE% yielded significant results, demonstrating a strong correlation with the experimental data (*p* < 0.05) (Table 1a). This conclusion is supported by a high coefficient of determination (*R* ^2^) of 99%, reflecting a strong correspondence between the experimental results and the model. Additionally, the adjusted *R* ^2^ (0.96) and predicted *R* ^2^ (0.88) values further affirm the quadratic model's effectiveness in accurately representing the data (Table 1a). The lack of fit was found to be insignificant (*p* > 0.05), confirming the accuracy and reliability of the quadratic model in predicting EE% values (Table 1a). The lack of fit test's *F*‐value indicated that the lack of fit was not significant compared to pure error, underscoring the adequacy of the developed second‐order polynomial model in agreement with the experimental data. Table 1b illustrates the variance analysis of encapsulation efficiency (EE%), highlighting significant linear relationships between the independent variables (A, B, and C) and the dependent variable (EE%). Specifically, the concentrations of phosphatidylcholine/cholesterol (A), Tween 80 (B), and green tea extract (GTE) (C) demonstrated statistically significant influences on EE% (*p* < 0.05), as shown in Table 1b.

**TABLE 1b:** Encapsulated samples of GTE with nanoliposome carriers.

Sample	Phosphatidylcholine: cholesterol (%)	Tween 80 (%)	GTE concentration (ppm)	Mean of loading capacity (%)	Encapsulation efficiency (%)	Standard deviation
T_1_	1:1	0.6	1000	6.307^hi^	56.522	0.120
T_2_	1:1	0.35	700	7.163^f^	62.848	0.118
T_3_	1:2	0.1	700	6.792^g^	60.475	0.136
T_4_	2:1	0.1	400	8.585^c^	77.926	0.102
T_5_	1:1	0.6	400	8.159^d^	73.259	0.108
T_6_	2:1	0.1	1000	6.022^jk^	53.224	0.124
T_7_	1:2	0.1	700	7.342^f^	64.772	0.088
T_8_	1:2	0.35	700	7.326^f^	65.150	0.096
T_9_	2:1	0.6	700	8.045^d^	71.476	0.207
T_10_	1:1	0.1	400	8.498^c^	75.541	0.212
T_11_	1:2	0.6	400	8.065^d^	71.879	0.222
T_12_	1:2	0.6	1000	6.002^jk^	52.598	0.038
T_13_	2:1	0.35	700	8.197^d^	73.523	0.1446
T_14_	1:2	0.1	1000	5.736^l^	50.569	0.123
T_15_	1:1	0.1	1000	5.731^l^	50.518	0.219
T_16_	1:1	0.6	700	7.612^e^	68.237	0.049
T_17_	2:1	0.35	1000	6.232^ij^	54.820	0.155
T_18_	1:2	0.1	400	7.625^e^	68.747	0.0551
T_19_	1:1	0.35	400	9.167^b^	82.879	0.190
T_20_	1:2	0.35	400	8.981^b^	80.748	0.143
T_21_	1:1	0.1	700	7.108^f^	62.689	0.083
T_22_	1:1	0.35	1000	5.937^kl^	53.209	0.114
T_23_	2:1	0.6	700	7.721^e^	69.173	0.101
T_24_	2:1	0.35	400	9.755^a^	93.480	0.167
T_25_	2:1	0.6	1000	6.473^h^	59.994	0.125
T_26_	1:2	0.6	1000	6.015^jk^	53.971	0.080
T_27_	2:1	0.6	400	9.593^a^	86.698	0.137

*Note:* Results are mean ± standard deviation. Different letters in the same row indicate that values differ significantly (*p* < 0.05). The device was set to a mean viscosity of 1.0200 and a mean refractive index of 1.335.

The optimization was carried out using three independent variables: (A) phosphatidylcholine:cholesterol ratio, (B) Tween 80 concentration (% w/v), and (C) GTE concentration (ppm), as shown in Tables 1a and 1b. The experimental design followed a Box–Behnken model under response surface methodology (RSM). In this study, “phosphatidylcholine” refers to soy lecithin (Lipoid S75) containing 80% phosphatidylcholine, and the lipid ratios listed in Tables 1a and 1b reflect the proportion of phosphatidylcholine (as lecithin) to cholesterol used in each formulation. The Tween 80 concentration was varied as a surface‐active stabilizer, while the GTE concentration represented the active loading variable in the system.

**FIGURE 1:**
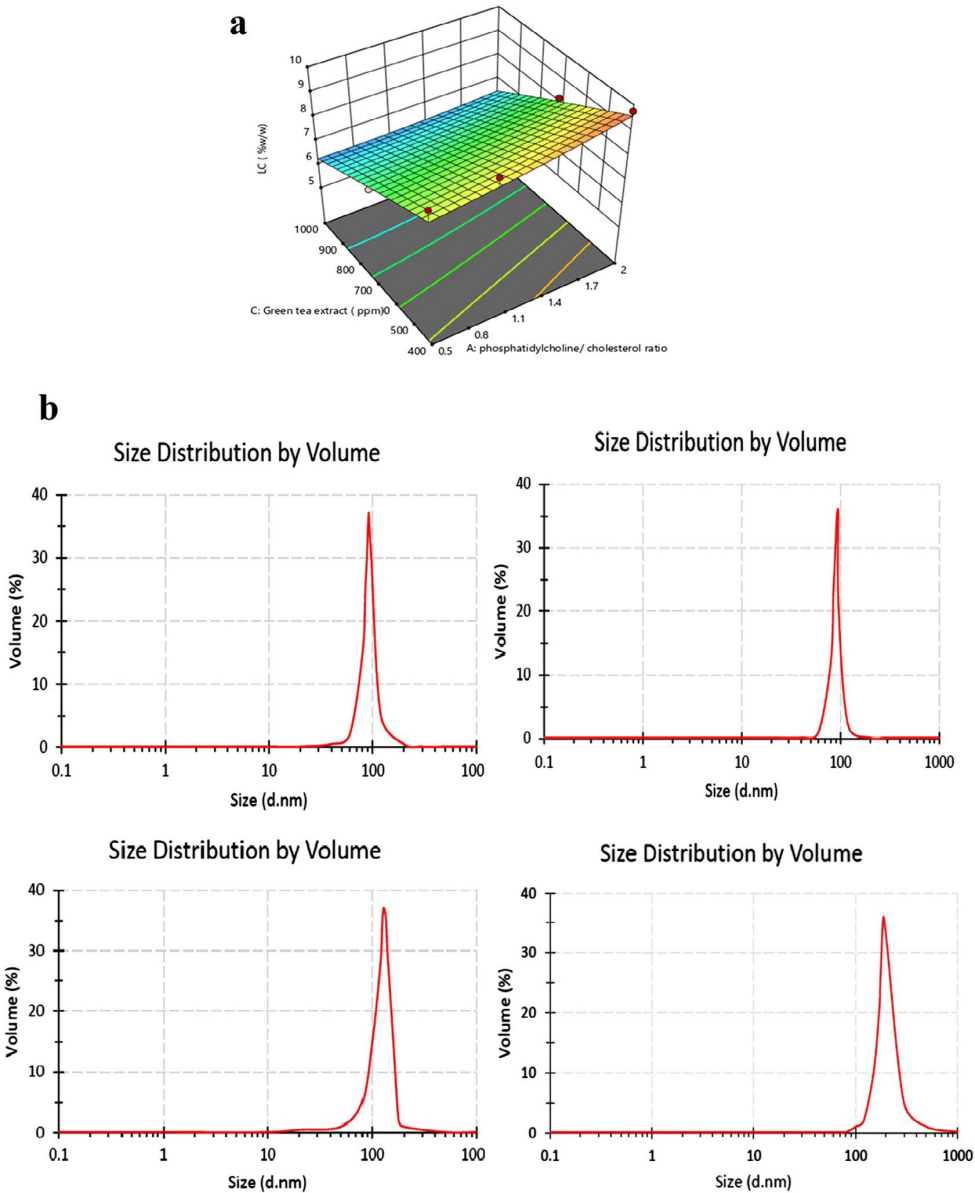
(a) Response‐level chart of phosphatidylcholine and GTE concentrations effects on encapsulation efficiency, (b) Particle size distribution of nanoliposome containing green tea extract on (1) day 1, (2) day 30, (3) day 60, (4) day 90.

The measured EE% values ranged from 50.518 ± 0.219 to 93.480 ± 0.167, suggesting that the addition of cholesterol and phosphatidylcholine to the liposomal membrane markedly affected the physicochemical characteristics of the liposomes. Research conducted by Lu et al. (2011) assessed the EE of GTE within liposomes utilizing a thin‐film ultrasonic dispersion method at various extract‐to‐lecithin ratios, resulting in EE values between 45% and 61.52%. As depicted in Figure 1a, a positive correlation was observed between the concentration of phosphatidylcholine and EE%, while an increase in GTE concentration had an adverse effect on EE%, resulting in a decrease. Figure 1a presents the response surface plots that illustrate the impact of varying concentrations of phosphatidylcholine and GTE on encapsulation efficiency (EE%). The data indicate that increasing the concentration of phosphatidylcholine correlates with an enhancement in EE%, whereas an increase in GTE concentration leads to a reduction in EE%. Similar observations were made by Takahashi et al. (2007), who noted that EE increased with lecithin concentrations up to 10% when utilizing a mechanical method for liposome formulation. In a related study, Lu et al. (2011) reported an improvement in EE as the ratio of tea polyphenols to lecithin was modified from 1:3 to 1:9, implying that lower levels of phosphatidylcholine may limit the availability of liposome vesicles required for effective polyphenol encapsulation. Additionally, Khosravi‐Darani et al. (2016) found that higher proportions of phosphatidylcholine enhance EE in liposomes containing Zataria multiflora Boiss essential oil. In contrast, Naghavi et al. (2016) observed a decline in EE with increased GTE concentrations, which they attributed to heightened negative charges and repulsive interactions, resulting in larger particle sizes and less stable liposomes. To optimize the encapsulation efficiency of GTE in nanoliposomes, it is essential to consider factors such as the phosphatidylcholine to cholesterol ratio, Tween 80 concentration, GTE concentration, and the method of preparation. The EE% is a pivotal factor influencing the loading capacity and overall efficacy of GTE nanoliposomes as a delivery system. Among the formulations tested, Sample T25 (PC:CH 2:1, Tween 80 0.6%, GTE 1000 ppm) was selected as the optimal formulation for detailed characterization due to its balance of encapsulation efficiency (60.09%), low average particle size (99.2 ± 0.34 nm), and stable zeta potential (~−30 mV), despite other formulations achieving higher EE values.

### 3.2 Particle Size Analysis

The particle size distribution of the GTE nanoliposome formulation over a 90‐day observation period is depicted in Figure 1b. This figure reveals a singular, well‐defined peak at each time interval, signifying a consistent droplet size distribution within the nanoliposome formulation. Such uniformity implies that the nanoliposomes preserved a stable particle size throughout the study, with no indications of diverse particle populations or aggregation.

The noted consistency in particle size distribution reflects minimal variation, suggesting that the differences in droplet sizes are limited and closely aligned with the actual droplet dimensions. This degree of uniformity is vital for evaluating the stability of liposomal formulations, as it indicates a reliable structure and composition, which are crucial for maintaining stability in both biological and food systems (Naghavi et al. 2016).

Table 2a provides a summary of the average particle diameter and polydispersity index (PDI) for the nanoliposome formulations. The optimal average particle size of 99.2 ± 0.34 nm suggests advantageous stability within food systems (Sheybani et al. 2023b). These results are consistent with findings from Lu et al. (2011), which also documented the formation of small, emulsion‐sized droplets.

**TABLE 2a:** *Z*‐average and PDI of GTE nanoliposome.

	Day 1	Day 30	Day 60	Day 90
Z‐average (nm)	99.2 ± 0.34^a^	99.2 ± 0.34^a^	99.2 ± 0.34^a^	99.1 ± 0.34^a^
Intercept	0.908 ± 0.06^a^	0.942 ± 0.06^a^	0.912 ± 0.06^a^	0.936 ± 0.06^a^
With (nm)	20	19	30	56
PDI	0.42 ± 0.05^a^	0.43 ± 0.05^a^	0.47 ± 0.05^a^	0.47 ± 0.05^a^

*Note:* The data are presented as mean ± standard deviation, and shared lowercase letters within each column indicate non‐significance at the 5% level.

The ANOVA analysis indicated that there was no statistically significant difference (*p* > 0.05) in the average particle size of liposomes containing GTE throughout a three‐month storage duration. This consistency in particle size may be linked to the pH‐dependent characteristics of the liposomes, which play a crucial role in their physicochemical stability. Comparable findings have been documented in previous research; for instance, Farshchi et al. (2021) observed that an increase in phospholipid concentration could result in a marginal rise in particle size in nanoliposomes that encapsulated ascorbic acid. Furthermore, Amiri et al. (2019) reported achieving optimal nanoparticle sizes between 82 and 88 nm when utilizing nanoliposomes for plant weed applications, which aligns with the outcomes of the current study. The existence of smaller particle spins or twins within the nanoliposome framework indicates a more restricted particle size distribution, reflecting a more uniform colloidal system.

In the context of nanoliposome research focused on encapsulating polyphenolic compounds, Zou et al. (2014) achieved a particle size of 66.8 nm for nanoliposomes infused with green tea polyphenols using an 8:1 phospholipid‐to‐polyphenol ratio, employing a combination of ethanol injection and microfluidization techniques. Similarly, Gülseren and Corredig (2023) fabricated nanoliposomes loaded with green tea polyphenols, attaining a particle size of 90 nm using soy phosphatidylcholine in conjunction with a high‐pressure homogenization technique.

Previous studies have highlighted the significant impact of liposome composition on average particle size. Gibis et al. (2012) reported that the size variation of liposomes depends on the nature of the encapsulated substances. In line with these findings, our study noted that the average particle size of liposomes containing GTE exceeded that of liposomes without GTE. This variation may be due to the accommodation of phenolic compounds within the bilayers of lipids and/or within the liposome core, potentially through hydrogen bonding interactions between the phenolic compounds and lipid molecules.

In this study, the PDI was found to be 0.42, indicating a consistent and narrow distribution, which is characteristic of successful nanoliposome production. A lower PDI denotes greater uniformity in particle size distribution, which contributes to a more homogeneous system resistant to phenomena such as Ostwald ripening. The results obtained in this study are generally consistent with those of Gibis et al. (2012), who also found that liposome size is influenced by the encapsulated bioactive components. This could be explained by the hydrogen interactions between the polar head group phospholipids and the phenolic compounds of GTE, as well as the hydrophobic interactions between the fatty acid tails of lipids and hydrophobic sections of phenolic compounds.

Throughout the storage period, the PDI for nanoliposomes containing GTE remained below 0.5, indicating a stable and uniform size distribution. Studies have demonstrated that varying concentrations of phosphatidylcholine and cholesterol during the synthesis of plant extracts can yield nanoparticles with PDIs as low as 0.3, reflecting uniform sizes. Conversely, a PDI above 0.7 indicates a highly varied size distribution, underscoring the importance of achieving a low PDI to ensure uniformity and stability in nanoparticle systems. This is supported by research indicating that PDI can be optimized by manipulating lipid composition and other synthesis parameters (Jahanfar et al. 2021).

### 3.3 Zeta Potential Analysis

**FIGURE 2:**
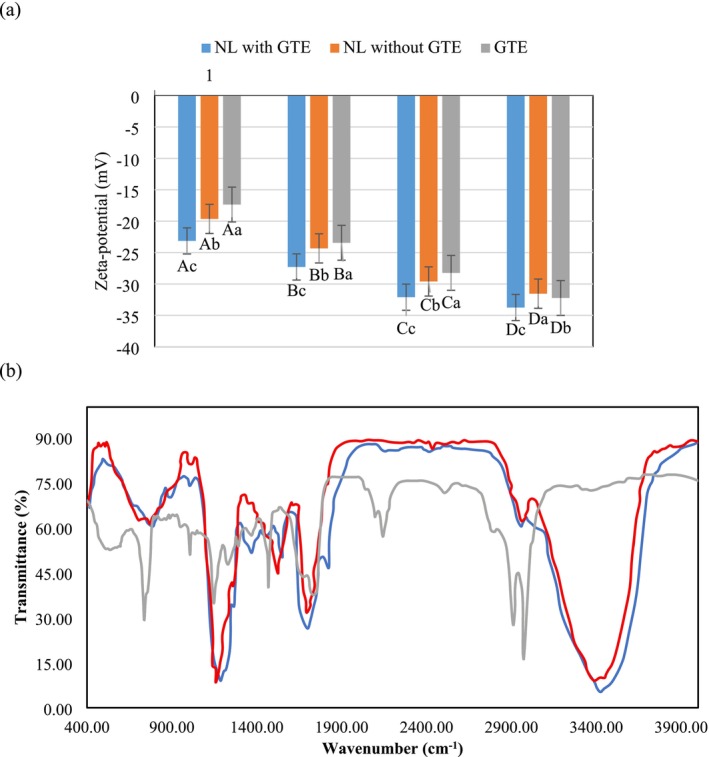
(a) Zeta potential during maintenance. (b) FTIR graphs of GTE‐free nanoliposome formulated by a combination of lecithin: cholesterol at a ratio of 40:20 (red), nanoliposome containing GTE after preparation (blue), and after 30 days (gray). Small non‐equivalent letters indicate significant differences in the values during the observed retention time (*p* ≥ 0.05). The non‐equivalent large letters indicate significant differences between each time point over the retention period (*p* ≥ 0.05).

Observations from zeta potential measurements for nanoliposomes encapsulating GTE, as depicted in Figure 2a, revealed a negative surface charge. The incorporation of GTE into the nanoliposomes resulted in an increase in the zeta potential, suggesting that the presence of GTE influences the surface charge, which may affect the stability and interactions within the liposomal system. An increase in cholesterol content was found to elevate the zeta potential levels. Cholesterol, being a nonpolar molecule, facilitates the formation of hydrogen bonds between the choline group in phosphatidylcholine and the hydroxyl group at the cholesterol head when the particle carries a negative charge. This interaction causes the choline group to acquire a positive charge within the membrane, while the phosphatidyl group retains a negative charge on the particle surface, thereby creating an electrostatic shield between them. Shah et al. (2020) concluded that cholesterol is frequently used in liposomal formulations to enhance their stability.

The accumulation of cholesterol within the lipid bilayers of liposomes plays a critical role in enhancing their structural stability and reducing functional deficiencies. However, formulations containing both cholesterol and lecithin introduce a substantial negative charge to the system, which can extend the shelf life of the liposome. Nonetheless, excessive cholesterol may increase membrane rigidity, potentially hindering the effective delivery of liposomes to target cells. Malheiros et al. (2012) reported that increasing cholesterol content in liposomes containing niacin raised the zeta potential from –55 to –64 mV. Similarly, Liu et al. (2012) employed ultrasonic film dispersion techniques to formulate green tea liposomes aimed at improving the bioavailability of tea polyphenols, achieving a zeta potential of –67.2 mV.

Lu et al. (2011) further demonstrated that increasing the cholesterol molar ratio to 0.75 in DPPC liposomes adjusted the zeta potential from +5 mV to −5.7 mV. In contrast, Gaur et al. (2016) observed that a higher cholesterol concentration of 16 μM did not significantly alter the zeta potential in ibuprofen‐containing liposomes.

Additional studies on liposomes incorporating phenolic compounds have reported a range of zeta potential values. For example, Lu et al. (2011) documented a zeta potential of −67.2 mV; Zou et al. (2014) reported −16.6 mV, and Gülseren and Corredig (2023) identified a zeta potential of −12 mV in nanoliposomes containing GTE polyphenols. These variations are likely due to the different properties of the phospholipids used in these studies. Generally, zeta potential measurements offer valuable insights into the surface charges and interactions within nanoliposomal systems encapsulating bioactive compounds such as GTE. The inclusion of cholesterol plays a significant role in modulating the zeta potential and enhancing the stability of liposomes. However, it is essential to achieve an optimal balance to ensure effective delivery of liposomes to target cells (Eskandari et al. 2021).

### 3.4 Intermolecular Interactions Analysis

FTIR spectroscopy was employed in this study to probe intermolecular interactions and assess the stability of GTEs encapsulated within nanoliposomes. This method is effective in identifying spectral shifts, band broadening, and peak intensity changes, all of which provide insight into the molecular interactions among the hydrophilic components of GTEs, empty nanoliposomes composed of phospholipids, and nanoliposomes encapsulating GTEs over periods of one day and one month. The objective of the study was to evaluate the stability of the encapsulated compounds during storage, and the FTIR analysis confirmed the successful encapsulation of GTE within the nanoliposomes, as depicted in Figure 2b. The analysis of pure lecithin and cholesterol revealed distinct spectral features corresponding to various functional groups. In lecithin, symmetric and asymmetric stretching vibrations of the CH_2_ groups were observed at 2857 and 2921 cm^−1^, respectively, with bending vibrations of CH_2_ appearing at 1458 cm^−1^. The frequency at 1733 cm^−1^ corresponds to the stretching vibrations of the aliphatic ester (C=O) group, while peaks at 1370 and 1172 cm^−1^ are associated with the CO—O—C and PO_2_ functional groups, respectively. For cholesterol, characteristic peaks include asymmetric CH_2_ stretching vibrations at 2890 cm^−1^ and bending vibrations of the CH_3_ group at 1436 and 1327 cm^−1^. The stretching vibrations of the C—O—P functional group were also noted at 1217 cm^−1^. These findings provide a detailed molecular fingerprint of the liposome's core components (Gülseren and Corredig 2023). The FTIR spectrum of a nanoliposome formulation devoid of GTE showed peaks at 2972 and 2910 cm^−1^, indicative of symmetric and asymmetric stretching vibrations of the CH_2_ groups. Additional peaks at 1468 and 1146 cm^−1^ correspond to PO_2_ and asymmetric C—O—O—C stretching vibrations, respectively. The similarity of these peaks to those observed in pure lecithin and cholesterol suggests that the nanoliposome structure predominantly comprises these two components.

The GTE itself exhibited distinct spectral features, with a prominent peak at 3386 cm^−1^ attributed to OH vibrations typical of phenolic compounds and a peak at 2959 cm^−1^ indicating the presence of alkane functional groups. Additional peaks at 1693 and 1158 cm^−1^ correspond to the stretching vibrations of C=O and C—O—P functional groups, respectively, while peaks at 1524 and 1158 cm^−1^ represent the asymmetric and symmetric bending vibrations of CH_3_. The FTIR analysis of nanoliposomes containing GTE immediately after preparation revealed several molecular vibrations, including peaks at 3424 cm^−1^ indicative of C—H tensile vibrations typical of alkanes, suggesting the presence of hydrocarbon chains. Symmetrical bending vibrations of CH_3_ groups were evident at 1698 cm^−1^, and asymmetric tensile vibrations of PO groups were detected at 1186 cm^−1^. CH_2_ oscillating vibrations identified at 788 cm^−1^ further confirmed the presence of alkyl chains within the liposome structure. Importantly, the spectrum also displayed peaks at 1550 and 1368 cm^−1^, indicative of polyphenols from the GTE, confirming the encapsulation and structural integration of these compounds within the nanoliposomes. The absence of peaks typically associated with the free OH group in GTE suggests that these groups are likely involved in hydrogen bonding within the encapsulated state, reducing their availability for interaction with infrared radiation.

The comparative analysis between FTIR spectra of GTE‐containing and GTE‐free nanoliposomes reveals specific indicator peaks that confirm the successful incorporation of GTE. For example, peaks at 3424 cm^−1^ indicate the presence of the general OH group, while peaks at 2957 and 2416 cm^−1^ are characteristic of the C—H bond in alkanes. The peak at 1821 cm^−1^ corresponds to the stretching vibrations of the aliphatic ester group (O=C functional group), and the peak at 1004 cm^−1^ suggests a stretching of the O—C bond. Minor spectral shifts and displacements, such as those noted at 2972, 2910, 1738, and 1146 cm^−1^, underscore the successful encapsulation of GTE within the nanoliposomes. The presence of C=H stretching vibrations indicates hydrogen bond formation, which has been reported to enhance the thermal stability of liposomal formulations. The stability of the GTE within the nanoliposome system is further affirmed by the absence of new peaks and the persistence of existing peaks in the FTIR spectrum over a 30‐day storage period. This suggests that no significant chemical reactions occurred between the liposomal nanocarrier and the GTE, indicating that both the nanocarrier and the extract maintained their structural integrity without undergoing any chemical transformations. This observation is crucial for confirming the chemical stability of the encapsulated compounds, which is essential for preserving their biological activity and therapeutic potential over time (Gülseren and Corredig 2023).

### 3.5 SEM Analysis of Nanoliposomes Encapsulating GTE

**FIGURE 3:**
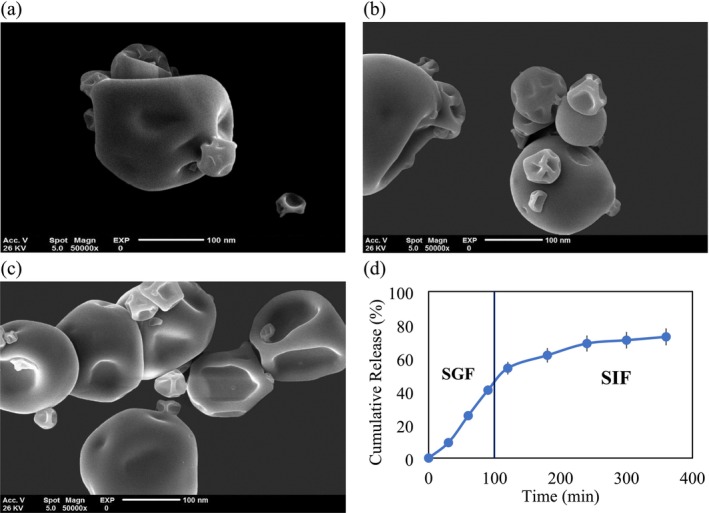
SEM observations of nanoliposome (a) containing GTE, (b) without GTE on day 1, and (c) containing GTE on day 90; (d) GTE release profile prepared in simulated gastric and intestinal fluids.

SEM analysis was employed to examine the morphology of liposomes loaded with GTE and to corroborate particle size distribution data obtained from DLS. As shown in Figure 3, the SEM images offer valuable insights into the solubilization behavior of hydrophilic compounds like GTE within the aqueous phase of lipid‐based nanocarriers. The SEM observations reveal that liposomes encapsulating GTE exhibit a more uniform and regular morphology compared to empty nanoliposomes. Specifically, Figure 3a shows GTE‐loaded nanoliposomes, which display a consistent spherical shape and smooth surface, while Figure 3b illustrates the morphology of empty nanoliposomes on day 1. These empty structures demonstrate a particle size of approximately 60 nm. After GTE integration, as shown in Figure 3a,c (day 1 and day 90, respectively), the particle size increased to around 100 nm, confirming the successful encapsulation of GTE.

The particle sizes observed through SEM align closely with those from DLS measurements, with SEM showing slightly larger values, which may indicate enhanced encapsulation efficiency and potentially greater bioavailability of GTE within the liposomal system. These measurements collectively validate the consistency and reliability of both SEM and DLS techniques for particle size assessment. Notably, Figure 3a,c, reveal distinct morphological changes between GTE‐loaded and empty nanoliposomes, with the GTE‐loaded liposomes showing a stable, spherical morphology that may contribute to increased encapsulation efficiency and improved structural integrity. Figure 3c also shows the morphology of GTE‐loaded liposomes after 90 days, where their stability and uniformity in structure are maintained, suggesting enhanced stability over time.

SEM analysis, conducted at a magnification of ×50,000, highlights the effects of GTE inclusion on the liposomal architecture, promoting the formation of stable spherical structures with a robust surface profile. This structural enhancement could facilitate effective targeted delivery of bioactive compounds, underlining the significance of GTE for the stability and efficacy of nanoliposomal formulations (Duhem et al. 2014; Heurtault et al. 2003).

### 3.6 Physical Stability and Size Dynamics of, and Encapsulation Efficiency of GTE Nanoliposomes Over Time

The physical stability of liposomes is a critical factor, as variations in particle size due to aggregation and agglomeration in buffer solutions can significantly affect their longevity (Eskandari et al. 2021). Research indicates that changes in particle size and distribution are often driven by the negative charges carried by phenolic compounds, which can lead to the destabilization and disintegration of nanoliposomes, ultimately resulting in their enlargement (Zou et al. 2014). The instability of liposomes is also influenced by specific formulation characteristics (Duhem et al. 2014). Particle size and distribution are essential parameters in characterizing liposomes, and maintaining these parameters consistently over time is a key indicator of stability. This study focused on assessing the physical stability of nanoliposomes and the efficacy of retaining encapsulated GTE over a 90‐day period.

As shown in Table 2b, the average particle size of nanoliposomes without GTE exhibited a slight increase from day 1 to day 30, likely due to nanoparticle aggregation. This aggregation is a common indicator of instability in colloidal systems, where attractive forces among particles cause them to cluster into larger aggregates. These composite units are formed through the coalescence of individual liposomes, primarily driven by van der Waals forces. As aggregation progresses, liposomes merge to form larger entities (Heurtault et al. 2003). In contrast, nanoliposomes containing GTE maintained a relatively stable particle size from the initial day throughout the study period, suggesting an initial aggregation of nanoparticles that dispersed over time (Shin et al. 2013). Further, Shin et al. (2013) investigated the stability of curcumin‐loaded nanoliposomes by examining changes in particle size at 4°C and 25°C. Nanoliposomes prepared using the dry thin layer method and stored at 25°C showed no significant change in size (*p* > 0.05), and those stored at 4°C exhibited minimal size variations on days 7 and 40. In contrast, nanoliposomes prepared by ethanol injection displayed a marked increase in size within the first five days of the 40‐day period at both temperatures (*p* < 0.05), followed by a decrease in the rate of particle size change toward the end of the study.

**TABLE 2b:** Stability of nanoliposome particle size (nm) over 3 months.

	Day 1	Day 30	Day 60	Day 90
Nanoliposomes containing GTE	99.2 ± 0.34^a^	99.2 ± 0.34^a^	99.2 ± 0.34^a^	99.2 ± 0.34^a^
Nanoliposome without GTE	99.0 ± 0.34^a^	99.6 ± 0.34^a^	99.5 ± 0.34^a^	99.5 ± 0.34^a^

*Note:* The dataset is composed of mean values ± standard deviations, with identical lowercase letters across each row indicating no statistical significance at the 5% level.

Table 2c presents the retention of encapsulation efficiency over 90 days of storage at 4°C. The EE% showed a minimal decrease from 60.09% on day 1 to 58.31% on day 90, with no statistically significant differences (*p* > 0.05). This finding confirms the high stability of the nanoliposome formulation and supports our claim of consistent encapsulation performance under refrigerated conditions.

**TABLE 2c:** EE% of GTE nanoliposomes over 90 days of storage at 4°C.

Time (days)	EE (%) ± SD
1	60.09 ± 0.13
15	59.64 ± 0.16
30	59.21 ± 0.15
60	58.79 ± 0.14
90	58.31 ± 0.17

*Note:* Values are mean ± SD (*n* = 3).

For nanoliposomes containing GTE, the encapsulation efficiency remained stable at a particle size of 99.2 nm from day 1 to day 90, indicating no significant alteration in size. This observation is notable given that liposomes are inherently thermodynamically unstable, with a natural tendency to fuse and release their encapsulated contents over time. The composition and amount of the active ingredient significantly impact particle size and, consequently, encapsulation efficiency. Incorporating the active substance both on the surface and within the liposomal structure enhances the concentration of the phospholipid membrane, resulting in a denser configuration. This densification helps maintain the physical stability of the nanoliposomes and ensures the retention of the encapsulated GTE over the 90‐day period. Figure 3c illustrates nanoliposomes exhibiting a bent conformation, with smooth surfaces in proximity. Notably, after 90 days of storage, the particle size remained at 99.2 nm, within the nanoscale range, without significant enlargement, indicating the successful preparation of the nanoliposomes. Additionally, the incorporation of cholesterol into the liposomal formulation enhances stability by inhibiting phospholipid phase transition (Malheiros et al. 2012), thereby contributing to the sustained integrity of the nanoliposomes.

### 3.7 Antioxidant Efficiency Assessment of GTE and Nanoliposome Containing GTE

Certain assays, such as the free radical scavenging test, are essential for evaluating the capacity of antioxidants to neutralize free radicals. Free radicals are highly reactive species capable of causing damage to lipoproteins, polyunsaturated fatty acids, DNA, amino acids, proteins, and sugars in both biological and nutritional contexts (Klančnik et al. 2009). In this study, the antioxidant efficacy of both free and encapsulated GTE was assessed using the DPPH (2,2‐diphenyl‐1‐picrylhydrazyl) assay, a widely accepted method for measuring the free radical scavenging activity of plant‐derived compounds. The results of this study demonstrate that encapsulation significantly enhances the antioxidant properties of GTE (Table 2d). These findings are consistent with those of Niu et al. (2012), who investigated the thermal stability and DPPH inhibition capacity of encapsulated curcumin. In their research, free curcumin showed a DPPH inhibition rate of 18.8%, whereas encapsulated curcumin exhibited a higher inhibition rate of 29.3% at 25°C. However, these results contrast with the findings of Zou et al. (2014), who studied tea polyphenol‐encapsulated nanoliposomes produced using ethanol injection and recycling techniques. Zou et al. (2014) reported that both polyphenol solutions and encapsulated tea polyphenols exhibited concentration‐dependent free radical scavenging activities. Notably, they did not observe a significant difference in DPPH free radical inhibition between the same concentrations of polyphenols before and after encapsulation.

**TABLE 2d:** The total phenolic content analysis and antioxidant activity in GTE and nanoliposome containing GTE.

	Total phenolic content (mg_GAE_ /g_GTE_)	IC_50_
GTE	31.87 ± 1.4^a^	13.52 ± 0.4^a^
nanoliposome containing GTE	78.97 ± 1.4^b^	1.16 ± 0.5^b^

*Note:* The dataset is composed of mean values ± standard deviations, with identical lowercase letters across each row indicating no statistical significance at the 5% level.

As shown in Table 2d, the average total phenolic content in the GTE and in nanoliposomes containing GTE was quantified as 31.87 and 78.97 mg of gallic acid equivalents (GAE) per g of extract, respectively. This significant increase in antioxidant activity following encapsulation into nanoliposomes is further evidenced by the reduction in the IC_50_ (the concentration required to inhibit 50% of free radicals) of the nanoliposome‐encapsulated GTE to 1.16 μg/mL. These results suggest that encapsulation not only preserves but also potentially enhances the antioxidant capacity of GTE, highlighting the advantages of nanoencapsulation in improving the bioavailability and efficacy of bioactive compounds.

### 3.8 In Vitro Release Kinetics Study

The study investigated the cumulative release of lecithin and phosphatidylcholine in SGF and SIF to evaluate the Alg‐CS complex layer deposited by electrostatic attraction. The release behavior over time exhibited a biphasic profile, characterized by an initial burst release phase during the first two hours in SGF, followed by a slower, sustained release phase over four hours in SIF. The duration of gastric emptying for a consumed meal is approximately 112 min, while the transit time within the small intestine ranges from 180 to 360 min (Cann et al. 1983; Davis et al. 1986). Experimental procedures were conducted under controlled conditions that simulated the gastric environment for 2 h, followed by a simulated intestinal environment for 6 h. Following a 2‐h incubation period in SGF, the release rate of GTE encapsulated within nanostructured lipid carriers (NLCs) reached 54%. This release rate increased to 73% after an additional 6‐h incubation in SIF, as shown in Figure 3d. The initial rapid release observed during the SGF phase, followed by a decline, suggests a release pattern governed by SGF‐related kinetics. Rehman et al. (2021) reported that NLCs exhibit a shell‐like structure enriched with GTE due to its homogeneous dispersion within the lipid phase after high‐pressure homogenization. The initial burst release observed within the first 30 min is likely due to the affinity of GTE for the nanoliposome surface, facilitating rapid absorption (Umakoshi 2004). The experimental results indicated a statistically significant decrease in the release rate from nanoliposomes in the SGF environment over time (*p* < 0.05). The presence of a regular lipid network contributes to the efflux of bioactive compounds, leading to a reduction in coverage percentage and a subsequent rapid release. The release mechanism of GTE in SGF is hypothesized to result from the immediate disintegration of the extract once adsorbed onto the nanoparticle surface. During dispersion, the solubility of the lipid component in the dispersing medium increases, enhancing the concentration of bioactive compounds at the nanoparticle level. The rapid release observed in the initial hours is likely associated with the swift disintegration of the extract within lipids under acidic conditions. Incorporating liquid lipids into the NLC formulation enhances the initial release rate (Dumont et al. 2019).

The sustained release of bioactive compounds beyond 1 h of digestion is attributed to their gradual liberation through successive layers of the coating, traversing the hydrocarbon section and encapsulated molecules. It has been observed that many liposomes without protective coatings remain relatively stable under acidic conditions (Li et al. 2015). However, at pH levels below 6.5, the acidic environment can induce hydrolysis of saturated phospholipids, leading to liposomal instability. Consequently, liposomes undergo degradation via hydrolysis, which facilitates the release of encapsulated GTE through the formation of pores and ruptures within the lipid matrices (Lin et al. 2015). The slow release observed in the intestinal environment suggests that a significant portion of the GTE remains entrapped within the NLC structure. Over time, the extract migrates from the deeper layers to the surface of the NLCs, resulting in a gradual release. After 2 h, a modest release rate is observed, continuing until the end of the digestion period. This behavior is influenced by the intestinal conditions, where, after 6 h, the release level of GTE reaches 73%. The enhanced encapsulation efficiency and reduced size of the NLCs contribute to increased release rates, amplifying the propulsion force and surface‐to‐volume ratio (Agnihotri et al. 2022). The release kinetics of nano‐formulated bioactive compounds in simulated intestinal environments mirrored the patterns observed under gastric conditions. The findings of this study, which demonstrate an initial rapid release phase under gastric conditions followed by sustained release in the lower intestinal tract, are consistent with the results reported in other studies in the field (Rashidi et al. 2022; Garcia‐Campayo et al. 2018; Wu et al. 2016). Nanoliposomes consistently exhibited behavior that contributed to the formation of a robust protective layer on the nanoliposome surface. This layer acts as a barrier, protecting the encapsulated bioactive compounds from the external environment and reducing direct interaction with the complex milieu of the stomach and intestines. The release dynamics of bioactive compounds from nanoliposomes encapsulating GTE are a multifaceted process that may involve release, degradation, and decomposition mechanisms, or a combination thereof. To better understand and predict the release and breakdown of these bioactive compounds from NLCs, various mathematical models have been utilized. These models provide a comprehensive understanding of the controlled release of active substances from the encapsulating matrix. In this study, the release behavior of GTE in SGF and SIF was analyzed using several mathematical modeling approaches, including zero‐order, first‐order, Korsmeyer–Peppas, and Higuchi models. The data analysis from the NLC release profiles was based on these models, as detailed in Table 3. The selection of the most appropriate model to describe the release kinetics of GTE from NLCs was based on the model with the highest coefficient of determination (*R* ^2^) value, indicating its predictive accuracy. The results highlighted that the Korsmeyer–Peppas model exhibited an excellent fit with the GTE release data, as evidenced by a near‐perfect *R* ^2^ value of 0.999. This high level of congruence suggests that the Korsmeyer–Peppas model effectively captures the complex release mechanisms of GTE from NLCs, underscoring its utility in predicting the release behavior of encapsulated bioactive compounds in various physiological environments.

**TABLE 3:** Release parameters of GTE from nanoliposomes in SGF and SIF.

Kinetic model	*R* ^2^	*K*	Simulated environment
Zero order	0.834	0.68	SGF
Zero order	0.647	0.21	SIF
First order	0.915	1.25	SGF
First order	0.932	1.69	SIF
Higuchi	0.906	0.25	SGF
Higuchi	0.975	0.62	SIF
Korsmeyer–Peppas	0.999	1.66	SGF
Korsmeyer–Peppas	0.995	1.51	SIF

## 4 Conclusion

This study demonstrated the successful encapsulation of GTE in liposomes using a thin‐film ultrasonic dispersion method, achieving optimal conditions with a tea polyphenol to lecithin ratio of 0.125:1, a lecithin to cholesterol ratio of 4:1, and PBS at pH 6.62. The resulting liposomes exhibited an encapsulation efficiency of 60.09%, and this formulation—optimized via Response Surface Methodology—was selected due to its desirable balance between EE, particle size, and zeta potential.

Green tea's diverse health benefits, including its antioxidant, antibacterial, antiviral, anti‐inflammatory, and anticancer properties, are largely attributed to its rich polyphenolic content, particularly EGCG. Encapsulation in liposomes was employed to protect these polyphenols from environmental degradation, enhance their bioavailability, and ensure controlled release. The study's findings affirm that encapsulation improves the stability and antioxidant properties of GTE, making it a viable strategy for enhancing the functional properties of green tea polyphenols in various applications, including food preservation and therapeutic use. The release kinetics of the encapsulated GTE followed a biphasic pattern, with an initial burst release phase observed in SGF and a subsequent sustained release phase in SIF. The Korsmeyer–Peppas model provided the best fit for describing the release behavior, indicating a complex mechanism involving both diffusion and matrix erosion. The stability of the nanoliposomes was further evidenced by their consistent particle size and encapsulation efficiency over a 90‐day storage period, highlighting the robustness of the liposomal formulation in preserving the bioactivity of GTE under simulated physiological conditions.

Overall, this study underscores the potential of liposome‐encapsulated green tea polyphenols as effective antioxidant delivery systems, capable of maintaining stability and enhancing bioavailability. The findings contribute to the broader understanding of using liposomal nanocarriers for the delivery of bioactive compounds, offering insights for future research into optimizing encapsulation techniques and formulations for enhanced therapeutic and nutritional applications. Unlike previous reports, our findings are supported by an integrated analysis combining kinetic modeling (*R* ^2^ = 0.999 for Korsmeyer–Peppas), structural FTIR confirmation over time, and a six‐fold improvement in antioxidant IC_50_ post‐encapsulation. These results provide a meaningful step forward in designing liposomal delivery systems for functional bioactives. While this study demonstrates the efficacy of nanoliposome‐based encapsulation for stabilizing and enhancing the antioxidant properties of GTE, certain limitations should be acknowledged. First, in vivo bioavailability, pharmacokinetics, and metabolic fate remain unexplored. Second, the study did not assess the interaction of the liposomal system with real food matrices, which may influence stability, release behavior, and sensory properties. Third, the scale‐up potential of the thin‐film ultrasonic method was not addressed, which may affect industrial applicability. Future research should investigate the in vivo efficacy of GTE‐loaded liposomes in animal or human models, their integration into food products, and their functional stability during processing and digestion. Additionally, exploring co‐encapsulation of synergistic bioactives (e.g., vitamins C or E) and investigating mucoadhesive or targeted delivery systems could further advance their nutraceutical and therapeutic potential.

## Author Contributions

Nadia Ahmadi performed tests and wrote the manuscript. Ladan Rashdi revised the draft; she is the corresponding author. Parham Joolaei Ahranjani reviewed and edited the manuscript.

## Conflicts of Interest

The authors declare no conflicts of interest.

## Data Availability

The current study is available from the corresponding author upon reasonable request.
